# Central Diabetes Insipidus in Children and Adolescents: Twenty-Six Year Experience from a Single Centre

**DOI:** 10.1155/2022/9397130

**Published:** 2022-03-08

**Authors:** Hüseyin Anil Korkmaz, Ritika R Kapoor, Jennifer Kalitsi, Simon JB Aylwin, Charles R Buchanan, Ved Bhushan Arya

**Affiliations:** ^1^Paediatric Endocrinology, Variety Club Children's Hospital, King's College Hospital NHS Foundation Trust, London, UK; ^2^Division of Paediatric Endocrinology, Department of Paediatrics, Izmir Dr. Behcet Uz Children's Hospital, Izmir, Turkey; ^3^Faculty of Medicine and Life Science, King's College London, London, UK; ^4^Faculty of Nursing, Midwifery and Palliative Care, King's College London, London, UK; ^5^Endocrinology, King's College Hospital NHS Foundation Trust, London, UK

## Abstract

**Introduction:**

Paediatric cohorts of central diabetes insipidus (CDI) have shown varying prevalence for different causes of CDI. The objective of this study was to determine the causes of CDI and long-term outcome in children and adolescents from a Tertiary Paediatric Endocrinology unit.

**Methods:**

The clinic database was searched to identify patients with CDI managed between 1993 and 2019. Relevant clinical information was collected from patient records.

**Results:**

A total of 138 CDI patients, median age 6 years (range <1–18) at presentation, were identified. Principal CDI aetiologies were craniopharyngioma (*n* = 44), acute central nervous system (CNS) insult (*n* = 33), germinoma (*n* = 15), postneurosurgery (indication other than craniopharyngioma and germinoma, *n* = 20), midline CNS malformation (*n* = 14), Langerhans cell histiocytosis (*n* = 5), and familial (*n* = 2). Idiopathic CDI in this cohort was infrequent (*n* = 5). Patients with CNS malformations/infections presented with CDI at a younger age compared to patients with CNS tumours (*p* < 0.0001). Five patients, initially presenting as idiopathic CDI, were subsequently diagnosed with germinoma after a median interval of 3.3 years. All patients with CDI related to craniopharyngioma and nearly all (87%) patients with CDI related to germinoma had concomitant GH, ACTH, and TSH deficiency. The majority of patients who manifested CDI due to acute CNS insult either deceased (30%) or had transient CDI (33.3%).

**Conclusion:**

Surgery for craniopharyngioma was the most common underlying aetiology of CDI with ubiquitous occurrence of panhypopituitarism in these patients. Manifestation of CDI in patients with acute CNS insult carries poor prognosis. We affirm that neuroimaging assessment in idiopathic CDI should be continued beyond 3 years from diagnosis as a significant number of patients exhibited progression of infundibular thickening 3 years post-CDI diagnosis.

## 1. Introduction

Central diabetes insipidus (CDI) is a heterogeneous condition characterized by polyuria (urine output >2 L/m^2^/24 hour) and polydipsia due to a deficiency of the hormone arginine vasopressin (AVP) [1, 2]. Principal CDI aetiologies include craniopharyngioma, germ cell tumors, midline central nervous system (CNS) malformations (septo optic dysplasia, congenital hypopituitarism, and holoprosencephaly), hypothalamic-pituitary injury from neurosurgery or head trauma, Langerhans cell histiocytosis (LCH), local inflammatory, autoimmune or vascular diseases, and genetic defects in AVP synthesis that are inherited in autosomal dominant or X-linked recessive traits [3, 4]. In a small proportion, no definite cause of CDI can be identified despite a thorough diagnostic workup, and these are considered idiopathic. In patients with apparently isolated CDI, differentiation between certain aetiological diagnosis such as LCH, dysgerminoma, and idiopathic can be difficult, except when dysgerminoma is associated with increased secretion of tumour markers in the CSF, rarely in the blood, or when tumour cells are observed in the CSF, and when another tumour is localized in the CNS, either in the pineal gland or, more rarely, the spinal cord. Extrapituitary involvement (bone, lungs, liver, skin, or mucosae) at diagnosis or during follow-up, and symmetrical and unifocal hypothalamic-pituitary lesions are suggestive of LCH [5].

A few groups have reported causes and long-term outcomes of paediatric CDI at a single institution or combined across multiple institutions [3, 4, 6]. These studies have shown a wide variation in the underlying causes of CDI, including prevalence of idiopathic diabetes insipidus. As CDI is a rare condition, there are few reports of large paediatric cohorts to effectively examine outcomes. Here, we report the causes of CDI in the largest paediatric cohort from United Kingdom reported to date as well as coexisting outcomes for anterior pituitary hormone deficiencies.

## 2. Materials and Methods

### 2.1. Patients

This was a retrospective single centre study performed at King's College Hospital (KCH), London, a tertiary paediatric endocrinology centre in south-east England with a catchment population of 3.5 million (population under 19 years old—approximately 800,000). We reviewed our database to identify all patients with a confirmed diagnosis of CDI seen at KCH from August 1993 through September 2019. The medical records of identified patients were reviewed to extract information including demographics, method of CDI diagnosis, surgical history, results of neuroimaging and anterior pituitary function evaluation at diagnosis and during follow-up, and clinical outcome.

### 2.2. Diagnosis and Classification of Central Diabetes Insipidus

The diagnosis of CDI was based on the clinical findings of polyuria and polydipsia, serum sodium >145 mmol/L, serum osmolality of >300 mOsm/kg of water, urine osmolality of <300 mOsm/kg of water, and an increase in urine osmolality in response to desmopressin. All diagnoses of CDI were made by a paediatric endocrinologist. MRI brain including gadolinium contrast-enhanced T1-weighted sequences in sagittal and coronal planes was obtained in all cases where CDI was persistent and/or no obvious cause of CDI was evident. The causes of CDI were classified as congenital malformations (septo optic dysplasia, holoprosencephaly, encephalocele, or congenital hypopituitarism), acquired (infiltrative or tumor, e.g., LCH, craniopharyngioma, germ cell tumor, other brain mass/cyst, infection/inflammation, or trauma), genetic or familial, or idiopathic. In the absence of a known predisposing condition and an abnormal initial pituitary MRI other than absence of T1 posterior pituitary bright spot (PPBS) at the time of CDI diagnosis, a patient was considered initially to have idiopathic CDI. Once an underlying diagnosis was identified, patients were assigned to the appropriate diagnosis category for further analysis.

### 2.3. Anterior Pituitary Function

All patients had evaluation of the anterior pituitary function by basal ± stimulated hormonal analysis at diagnosis and follow-up. Pituitary hormone deficiency diagnoses were based on a combination of clinical assessment and laboratory tests, including serum insulin like growth factor-1 (IGF-1) and provocative testing for GH deficiency, free thyroxine levels, and thyroid stimulating hormone (TSH) for TSH deficiency, low early morning cortisol or response to cosyntropin stimulation testing for ACTH deficiency, and gonadotropin and sex hormone levels for gonadotropin deficiency.

### 2.4. Statistical Analysis

Data were analysed using GraphPad Prism version 9.0.0 for macOS, GraphPad Software, San Diego, California USA, http://www.graphpad.com. Descriptive data and the included figures were obtained from GraphPad software. Continuous variables are given as medians (with interquartile ranges), and categorical variables are expressed as frequencies (with percentages). Medians were compared using the Mann–Whitney *U*-test. The data was assessed for normality using Shapiro–Wilk test. *P* values of ≤0.05 were considered statistically significant.

## 3. Results

One hundred and thirty-eight patients with a diagnosis of CDI were identified. The patients in our cohort were followed up for an median of 5.5 years (interquartile range 8.2 years) from diagnosis to the end of data collection for this study. For the 116 patients who had available information, 57 patients (49%) were diagnosed on the basis of a single measurement laboratory tests, while in the paediatric intensive care unit, recovering from cranial surgery, and 38 patients (32.8%) with nonfasting laboratory results in the context of a significant risk factor for CDI (e.g., known brain malformation, severe head injury, intracranial haemorrhage, or CNS infection). 12 patients (11.5%) were diagnosed by water deprivation test and 9 patients (7.7%) with a single measurement of basal laboratory tests.

**Figure 1:**
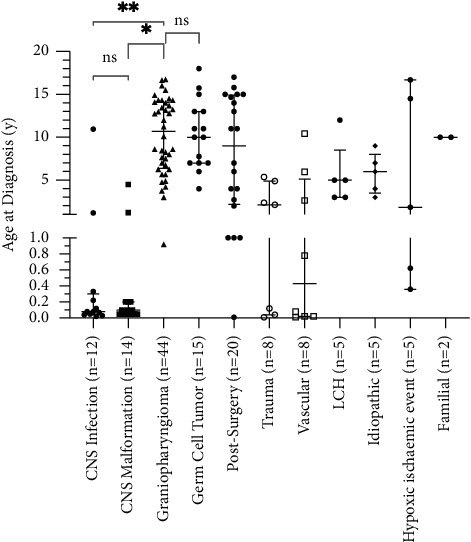
Median (with interquartile range) age at diagnosis of Central Diabetes Insipidus for different aetiologies (^*∗*^*p* < 0.0001; ^*∗∗*^*p* < 0.0001; CNS–central nervous system, LCH–Langerhans cell histiocytosis).

The aetiologies of CDI in our cohort of 138 patients (81 males) are shown in Table 1. The principal aetiologies were craniopharyngioma and/or its treatment (*n* = 44), germinoma and/or its treatment (*n* = 15), postneurosurgery (indication other than craniopharyngioma and germinoma, *n* = 20), midline CNS malformation (*n* = 14), and acute CNS insult (*n* = 33). Only 1 of 44 craniopharyngioma patients presented with symptoms of CDI, whereas the rest developed CDI postsurgery. Five patients had LCH, all diagnosed with biopsy of associated skin lesions. Further five patients had idiopathic CDI at last clinical assessment. Median age at CDI diagnosis for different aetiologies is shown in Figure 1.

**Figure 2:**
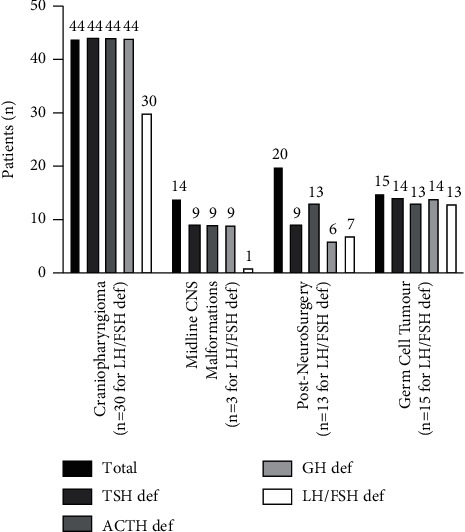
Coexistent anterior pituitary hormone deficiencies in patients with central diabetes insipidus (the number of patients who had reached age for definitive assessment for FSH/LH deficiency is mentioned separately in brackets; ACTH—adrenocorticotropin; FSH—follicle stimulating hormone; GH—growth hormone; LH—luteinising hormone; TSH—thyrotropin).

Figure 2 displays the accompanying anterior pituitary hormone deficiencies seen in our cohort of patients with CDI. All patients with CDI related to craniopharyngioma and nearly all (87%) patients with CDI related to germinoma had concomitant GH, ACTH, and TSH deficiency. In the postneurosurgery cohort (20 patients; for indication other than craniopharyngioma and germinoma), GH, TSH, and ACTH deficiencies were present in 6, 9, and 13 patients, respectively. In the midline CNS malformation group (14 patients), 9 patients each had concomitant GH, ACTH, and TSH deficiency.

Of the 15 germinoma patients with CDI, eight patients showed evidence of tumour on the first MRI after initial diagnosis of CDI, which was subsequently confirmed as germinoma by biopsy. Two germinoma patients developed CDI soon after the biopsy/tumour debulking. Five patients, initially presenting as idiopathic CDI, were eventually diagnosed with germinoma after a median interval of 3.3 years (range 0.5–4.3 years).

### 3.1. Central Diabetes Insipidus in Children with Acute Brain Insult (Figure 3)

**Figure 3:**
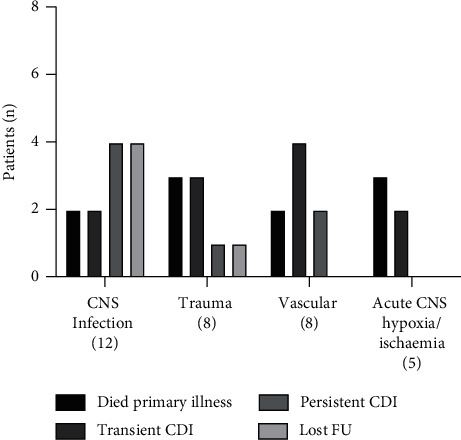
Outcome of patients with central diabetes insipidus (CDI) secondary to acute brain insult. (CNS—central nervous system; FU—follow-up).

Thirty-three patients (22.6%), aged 3 days to 16.7 years, developed CDI secondary to acute brain insult. Ten of these patients (30%) died after a median duration of 3 days (range 2 days to 6 weeks) after the diagnosis of CDI as a consequence of the underlying intracranial insult. In 11 (33.3%) patients, CDI was transient and resolved after a median duration of 6 months (range 6 days to 36 months). In 7 patients, CDI was persistent at last clinical review. In 5 patients, follow-up information was not available as they had been transferred to other healthcare facilities. In 7 patients with persistent CDI due to acute brain insult, 4 patients had additional TSH and ACTH deficiency, 1 patient subsequently developed precocious puberty, and 2 patients had no additional pituitary hormone dysfunction. Figure 3.

### 3.2. Outcomes of Those Initially Presented as Idiopathic CDI

Ten patients were diagnosed with CDI in the absence of any medical conditions known to be associated with CDI and had no known cause of CDI after their initial MRI and were initially considered idiopathic. Five (50%) had abnormal infundibular thickening. Four were later diagnosed with germinoma after a median follow-up of 3.3 years. One additional patient, who had absent PPBS but no infundibular thickening, also developed germinoma after 3.7 years of follow-up. The other five patients, one with infundibular thickening and four with absent PPBS, remain idiopathic with no underlying cause identified after median follow-up of 10 years.

## 4. Discussion

This study presents one of the largest cohorts of paediatric CDI patients in the literature and is the largest series on paediatric CDI from a paediatric tertiary endocrine centre in the United Kingdom. The results demonstrate that the underlying causes of CDI are significantly different across different age groups with CNS tumours accounting for majority of patients with CDI in older children and young adults.

Of interest, a larger proportion of patients in our cohort had CDI due to acute CNS insult than most previously published paediatric CDI cohorts [3, 6–9]. The authors believe this difference is due to our centre being a large regional centre for paediatric neurosurgery and brain trauma. Post-CNS insult CDI may result from inflammatory oedema around the hypothalamus or posterior pituitary or from direct damage to the paraventricular and supraoptic hypothalamic neurons, the pituitary stalk, or axon terminals in the posterior pituitary [10]. The development of CDI after CNS injury is a sign of severe brain damage and carries a poor prognosis with high mortality [11]. Approximately one-third of patients with CDI due to acute CNS insult in our cohort died after a median duration of 3 days from the diagnosis of CDI. There is scarce information in the literature regarding resolution of CDI in survivors of acute CNS insult. One-third of patients with CDI associated with acute CNS insult in our cohort had transient CDI, lasting a median duration of 6 months (range 6 days to 36 months). Prospective studies in the paediatric age group have shown that many of the endocrine abnormalities, including CDI, found in the first few months after acute brain insult may resolve; hence, recommendation that close endocrine surveillance should be followed for at least 1 year [12, 13].

Previous reports have clearly established that idiopathic CDI represents a small proportion of all children with CDI, and long-term surveillance of patients without a proven genetic cause is important [4]. Distinctively, our cohort presents a lower percentage of patients with idiopathic DI (3.6%) than many previously published studies. This is likely due to our cohort including patients with recognised predisposing conditions for CDI, rather than restricted to those with an initial presentation of polyuria and polydipsia. In contrast, our cohort reports a higher percentage of patients with craniopharyngioma (32%) than the average 22.5% (15–27%) from the other six paediatric CDI series (*n* = 34–147) [3, 4, 6–9]. It is noteworthy, that only one of 44 craniopharyngioma patients presented with symptoms of CDI, whereas the rest developed CDI postsurgery. Our observations are similar to those published by Bajpai et al. [7]. All 11 patients with craniopharyngioma in their study developed CDI after surgery. Few studies did not comment on this distinctive observation [4] whereas Di Iorgi et al. reported six patients with craniopharyngioma who presented with symptoms of CDI [14].

Of the 10 patients with initially idiopathic CDI, a cause was later identified in 5 patients. Although mild infundibular thickening was present throughout MRI surveillance in four patients, significant increase in infundibular thickening developed more than 3 years after the initial CDI diagnosis in four patients (including 1 patient with no initial infundibular thickening). Our findings highlight that despite no report of concerning MRI changes after 3 years of follow-up in two recent large series [4, 14], the latent period between infundibular thickening and diagnosis of germinoma is unpredictable, and long-term MRI surveillance of patients with idiopathic CDI is of paramount importance. Similar time intervals between onset of CDI and diagnosis of intracranial tumours have been described [15, 16].

Septo-optic dysplasia (SOD) was the underlying cause of CDI in eight patients (5.8%) in our cohort. Although SOD is the commonest anatomical CNS malformation associated with CDI, most SOD patients do not manifest CDI. In our data base, 8/68 SOD patients (11.8%) manifested CDI, findings which are not dissimilar to 16.8% prevalence in a recently reported large SOD cohort [17]. In this cohort of 8 SOD patients with CDI, coexistent GH, ACTH, and TSH deficiencies were present in 7, 7, and 8 patients respectively.

Remarkably, our entire cohort of craniopharyngioma patients with CDI had panhypopituitarism. Although the results give the impression that LH/FSH deficiency is less prevalent, this is certainly not the case as some children have yet to attain a pubertal age, and gonadotropin deficiency is difficult to diagnose in these cases. As Werny et al. grouped their cohort of craniopharyngioma patients with other acquired infiltrative and tumour causes, direct comparison of anterior pituitary deficiencies was not possible. In comparison, Tan et al. reported anterior pituitary hormone deficiency at latest follow up in 59-93% in a cohort of 85 craniopharyngioma patients managed during the same time interval (1998–2011) as our study, GH deficiency being the commonest [18].

There are limitations to this report that deserve consideration. Firstly, being a retrospective report, our study did not allow for rigorous prospective MRI evaluations as performed by Di Iorgi et al. [14]. Secondly, laboratory data from the time of diagnosis for few patients were missing either because the investigations were performed at another centre or were performed before 1999 when our institution started using the present laboratory records system. Thirdly, we were not able to perform genetic testing in all our patients that are classified as idiopathic CDI in the presence of lack of family history and absence of additional pituitary hormone deficiencies. Although they are unlikely to have a genetic aetiology for their CDI due to above mentioned reasons, there is a small possibility that some of these patients remain misclassified as idiopathic.

In conclusion, this is one of the largest single-centre cohort of patients with CDI described in the literature. The majority of patients who manifested CDI due to acute brain insult either deceased or had transient CDI, emphasising the need for careful endocrine reassessment in survivors. Idiopathic CDI in paediatric population was infrequent. We advocate the need for neuroimaging reassessments in idiopathic CDI beyond the recommended 3 years from diagnosis [4, 14] as a significant number of patients exhibited progression of infundibular thickening to germinoma 3 years post-CDI diagnosis.

## Figures and Tables

**Table 1:** Aetiology and age at diagnosis of central diabetes insipidus in 138 patients.

Diagnosis	No. of patients (%)	Median age at diagnosis (years)
Craniopharyngioma and/or its treatment	44 (31.6)	10.7
Germinoma and/or its treatment	15 (11.5)	10.5
Postneurosurgery (indication other than craniopharyngioma and germinoma)	20 (14.4)	9.0
Acute CNS insult		
CNS infection	12 (8.6)	0.08
Head trauma	8 (5.8)	2.1
Vascular lesion	8 (5.8)	0.4
Hypoxic ischaemic event	5 (3.6)	1.8
CNS malformation	14 (10.1)	0.1
Langerhans cell histiocytosis	5 (3.6)	5.0
Familial	2 (1.4)	10
Idiopathic	5 (3.6)	6.0

## Data Availability

Data sharing is not applicable to this article as no new data were created or analysed in this study.
